# [Retracted] SIRT1 activation by resveratrol reduces brain edema and neuronal apoptosis in an experimental rat subarachnoid hemorrhage model

**DOI:** 10.3892/mmr.2025.13603

**Published:** 2025-06-30

**Authors:** Cong Qian, Jianxiang Jin, Jingyin Chen, Jianru Li, Xiaobo Yu, Hangbo Mo, Gao Chen

Mol Med Rep 16: 9627–9635, 2017; DOI: 10.3892/mmr.2017.7773

Following the publication of the above article, a concerned reader drew to the Editor's attention that, regarding the western blots shown in Fig. 4A on p. 9632, there was a possible break in the continuity of the gel slice representing the β-actin bands in this figure, such that the protein bands were not shown consecutively as they would have appeared in the affected gel slice.

After having conducted an internal investigation, it became apparent that other western blots were potentially affected by the same issue (for example, the data shown in Fig. 4C). The Editor of *Molecular Medicine Reports* therefore shares the reader's concern that, beyond reasonable doubt, there were anomalies associated with the presentation of the western blot data in Fig. 4, and on the grounds of a lack of confidence in the presented data, the Editor has decided that the article should be retracted from the publication. The authors were asked for an explanation to account for these concerns, but the Editorial Office did not receive a reply. The Editor apologizes to the readership for any inconvenience caused, and we also thank the reader for bringing this matter to our attention.

